# Serum creatinine/cystatin C ratio as a muscle mass evaluating tool and prognostic indicator for hospitalized patients: A meta-analysis

**DOI:** 10.3389/fmed.2022.1058464

**Published:** 2023-01-09

**Authors:** Wen-He Zheng, Yi-Bing Zhu, Yan Yao, Hui-Bin Huang

**Affiliations:** ^1^Department of Critical Care Medicine, The Second People’s Hospital Affiliated to Fujian University of Traditional Chinese Medicine, Fuzhou, China; ^2^Department of Critical Care Medicine, Guang’anmen Hospital, China Academy of Chinese Medical Sciences, Beijing, China; ^3^Department of Critical Care Medicine, Beijing Tsinghua Changgung Hospital, School of Clinical Medicine, Tsinghua University, Beijing, China

**Keywords:** creatinine/cystatin C ratio, mortality, hospitalized, meta-analysis, sarcopenia

## Abstract

**Objective:**

Sarcopenia is a syndrome of decreased muscle mass and deficits in muscle strength and physical function. We aimed to investigate the relationship between creatinine/cystatin C ratio (CCR) and sarcopenia and the prognostic value of CCR in hospitalized patients.

**Materials and methods:**

We searched for relevant studies in PubMed, EMBASE, and the Cochrane Database up to August 25, 2022. Meta-analyses were performed to evaluate the relationship between CCR and skeletal muscle [computed tomography-assessed skeletal muscle (CTASM), muscle strength, and physical performance], prognosis and important clinical outcomes in hospitalized adults. The pooled correlation coefficient, the area under the receiver operating characteristic (ROC) curves, and hazard ratio (HR) together with their 95% confidence intervals (CIs) were calculated. We also conducted subgroup analyses to explore the sources of heterogeneity.

**Results:**

A total of 38 studies with 20,362 patients were eligible. These studies were of moderate to high quality. Our results showed that CCR was significant correlations with all CTASM types (Fisher’s *Z* ranged from 0.35 to 0.5; *P* values ranged from < 0.01 to 0.01), handgrip strength (Fisher’s *Z* = 0.39; 95% CI, 0.32–0.45; *P* < 0.001) and gait speed (Fisher’s *Z* = 0.25; 95% CI, 0.21–0.30; *P* < 0.001). The ROC curves suggested that CCR had good diagnostic efficacy (0.689; 95% CI, 0.632–0.746; *P* < 0.01) for sarcopenia. CCR can reliably predict mortality in hospitalized patients, which was confirmed by regression analysis of CCR as both continuous (HR 0.78; 95% CI, 0.72–0.84; *P* < 0.01) and categorical variables (HR 2.05; 95% CI, 1.58–2.66; *P* < 0.0001). In addition, less evidence showed that higher CCR was independently associated with a shorter duration of mechanical ventilation, reduced length of stay in the intensive care unit and hospital, less nutritional risk, and decreased complications in hospitalized patients.

**Conclusion:**

CCR could be a simple, economical, and effective screening tool for sarcopenia in hospitalized patients, and it is a helpful prognostic factor for mortality and other important clinical outcomes.

**Systematic review registration:**

https://inplasy.com/inplasy-2022-9-0097/, identifier INPLASY202290097.

## Introduction

Sarcopenia is traditionally been considered a syndrome characterized by reduced muscle mass, deficiencies in muscle strength, and impairments physical function (1). It has been thought that sarcopenia is more prevalent in old patients, especially those over 65 (2, 3). Sarcopenia, however, is common in hospitalized patients of all ages and is associated with various adverse outcomes, including impaired organ functions, infectious complications, prolonged length of stay (LOS) in intensive care unit (ICU) or hospital, and even increased mortality rates (4–7). Therefore, adequate body muscle reserve is crucial for hospitalized patients’ recovery and survival.

Previous indicators used to evaluate muscle reserve, such as anthropometrics, lab tests, subjective judgment, and body mass index (BMI), fail to reflect the patient’s body composition accurately (8). Clinicians may now directly measure muscle mass thanks to advances in imaging and software technologies (9, 10). In particular, computed tomography (CT), has been recognized as the gold standard for identifying skeletal muscle because it can accurately distinguish skeletal muscle and fat mass using a single cross-sectional slice at multiple body levels (11). However, high cost, radiological damage, and equipment unavailability limit the widespread use of these techniques (10, 11). In addition, these techniques are not conducive to continuous monitoring of muscle mass changes. As a result, there is an urgent need for other simple and inexpensive biomarkers to diagnose and monitor sarcopenia.

Serum creatinine and cystatin C are widely used in clinical practice to assess renal function. And the ratio of the two markers (serum creatinine/serum cystatin C) x100, known as CCR, has recently attracted interest. In 2016, Kashani et al. validated the correlation between muscle mass and CCR, which they defined as the “sarcopenia index” (12). Moreover, the authors found that CCR could predict hospital and 90-day mortality in patients who did not have acute renal damage. Since then, CCR has been increasingly used for critically ill patients (13, 14), the elderly (6, 7), organ transplant recipients (15, 16), and type 2 diabetic patients (17). However, substantial variation in study design, sample size, demographics, and muscle assessment among these studies lead to inconsistent results (6, 7, 12, 13, 18). Furthermore, there are no meta-analyses to examine the values of CCR on muscle mass measurement and prognosis in these patient populations.

Several studies on CCR in hospitalized patients have been published recently (14, 19–24). Therefore, with the power of meta-analysis, we aimed to perform a systematic review and meta-analysis of available published articles about hospitalization patients to investigate (1) whether CCR is a better and more accurate index of muscle mass, muscle strength, and gait speed; (2) the applicability of using CCR as a screening method for sarcopenia; and (3) the association of CCR with clinical outcomes (i.e., mortality, duration of mechanical ventilation, length of stay in ICU or hospital, and complications).

## Materials and methods

The systematic review was already registered in the International Platform of Registered Systematic Review and Meta-analysis Protocols database, and it is now available in its entirety on inplasy.com.^1^ It was carried out in accordance with the PRISMA (Preferred Reporting Items for Systematic Reviews and Meta-Analyses) guidelines (25; Supplementary file 1).

### Search strategy

We conducted a systematic search of relevant studies in PubMed, EMBASE, and the Cochrane Library from their establishment to August 25, 2022 (The last search date). Using a combination of MeSH and keywords, search terms included “creatinine,” “cystatin C,” “creatinine/cystatin C ratio,” “creatinine to cystatin C ratio,” “creatinine over cystatin C ratio,” “creatinine-to-cystatin C ratio,” and “sarcopenia index.” We restricted the language to English. Two authors (W-HZ and YY) independently imported the papers into Endnote X7 to exclude duplicate research and screen the literature (titles, abstracts, and full texts). We read meta-analyses, reviews, and comments to find more potential articles. The reference lists of the included full-text papers were also examined. We included the most recent published or reported data for republished studies. Disagreements were resolved through discussions between the two authors.

### Inclusion and exclusion criteria

We included articles investigating the correlation between CCR and CT-assessed skeletal muscle and the predictive prognosis value of CCR in hospitalized patients. The particular inclusion criteria were as follows, based on the PICOS (population, intervention, comparison, outcome, design) principle:

(1) adult (> 18 years old) hospitalized patients.
(2) evaluation of skeletal muscle amount (area) or quality (density) as determined by CT using any clear and objective methods.
(3) studies should report the correlation between CCR and CTASM or patient survival information.
(4) eligible studies had a cohort, case-control, or randomized controlled study design.

We excluded the studies that reported data without predefined outcomes and focused on animals or pregnant women. Studies available only in abstract form or meeting reports were also excluded.

### Data extraction

Two authors (W-HZ and YY) independently extracted data from included studies on the following items: first author, publication year, geographic location, study design, research period, population, sample size, demographic characteristics, disease severity, details on CT technique (muscle measured, CT-scan level), sarcopenia criteria and prevalence, outcomes of interest and methodological quality.

### Quality assessment

Two authors (W-HZ and YY) independently assessed the quality of each included study using the Newcastle-Ottawa Scale (NOS) for cohort studies (26). The NOS is divided into three domains depending on the cohort’s selection, the comparability of the groups, and the quality of the results. The included research was granted a maximum of one point for each item in the selection and outcome domains, and a maximum of two points for the comparability domain. The scale ratings ranged from 0 to 9, with 8 or 9 being categorized as good quality, 6 or 7 as moderate quality, and 5 or less as low quality. Disagreements were recognized and addressed through discussion.

### Statistical analysis

The primary outcome of the current meta-analysis was to investigate the suitability of CCR as a predictive mortality tool in hospitalized patients. To minimize potential interference factors, we only pooled the regression analysis findings of the included studies to investigate the link between CCR and mortality at the longest follow-up available. The HR in related studies were converted to their natural logarithms, and standard error (SE) values were determined using these logarithms and their respective 95% CI.

Secondary outcomes included associations between CCR and CTASM evaluation, muscle strength, gait speed, nutrition screening tool, or other clinical outcomes (i.e., duration of mechanical ventilation, length of stay in ICU or hospital, and complications). As to these outcomes, we conducted related meta-analyses individually for the various data reporting types as follows among the included studies. (1) For the studies that provided the correlation coefficient between CCR and predefined outcomes (i.e., CTASM, handgrip strength [HGS], gait speed [GS], and nutrition screening tool), we performed a meta-analysis by quantitatively summarizing the correlation coefficient statistic (r) estimates. Fisher’s Z and its SE were calculated using r and sample size (N) as follows: Zr = (1/2) [loge (1 + r) − loge (1 − r)], SEzr = 1/sqrt[N−3] (27). After appropriate transformation, we used the inverse variance-weighted approach to determine effect sizes and the associated 95% confidence intervals (CI). (2) For the studies reporting the diagnostic value of CCR for detecting sarcopenia, we pooled Area under the curve (AUC) values using the mean and standard error SE values and weighted them using the inverse-variance method (28). (3) We collected and pooled OR with 95% CI *via* the generalized inverse-variance method for studies that showed an association between CCR and sarcopenia using regression analysis. Unless otherwise noted in the above meta-analyses, we preferred the adjusted analysis results.

We used the *I*^2^ statistic to quantify heterogeneity (*I*^2^ < 50 and > 50% were classified as low and high heterogeneity, respectively) (29). When there was significant heterogeneity, a random-effects model was used; otherwise, a fixed-effects model was utilized (30). We then performed sensitivity analyses, removing one study at a time to demonstrate the impact of that study on the pooled effect estimates. Visually inspecting funnel plots for asymmetry was used to determine publication bias. Meta-analysis was conducted when data from at least 3 studies were available. *P* values of less than 0.05 were regarded as statistically significant. We utilized R version 3.6.2 for all statistical analyses in the current meta-analysis.

Subgroup analyses were performed to find the potential sources of heterogeneity on the following properties:

(1) Geographic location: Asian and other countries;
(2) Patient population: critical illness, cancer, medical, and surgery patients; and
(3) Gender (i.e., male and female).

## Results

### Study selection

**FIGURE 1:**
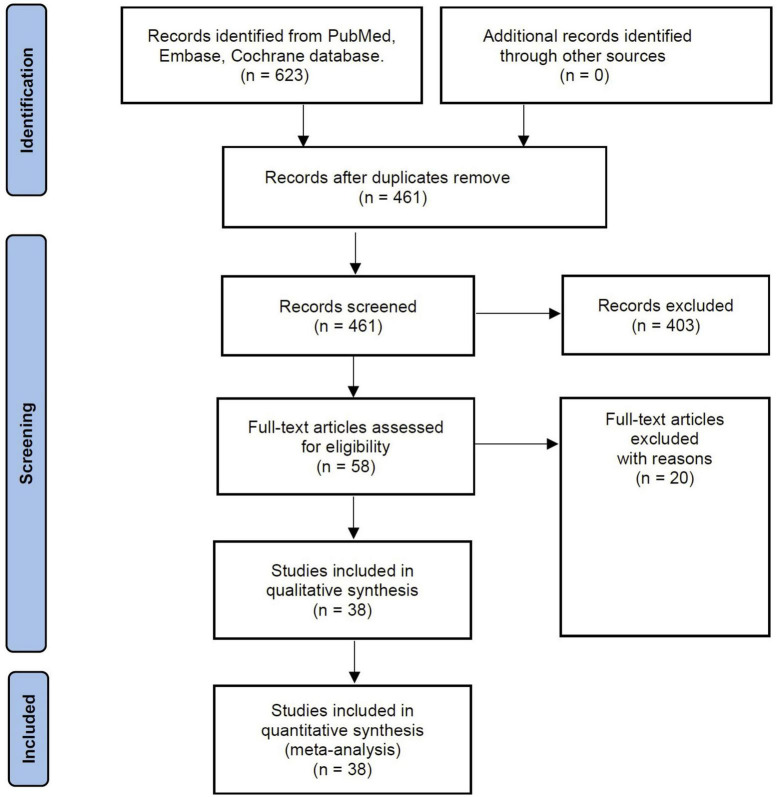
Flow chart of literature selection.

The comprehensive literature search yielded 213 studies. Following evaluation of the title, abstract, and full text, 38 papers involving 20,362 participants fulfilled the criteria for inclusion in the current systematic review (5–7, 12–24, 31–52). Figure 1 shows how the search strategy flows and the selection process.

### Study characteristics and methodological quality

Table 1 and Supplementary file 2 present the characteristics of the eligible studies. All these observational studies were published between 2016 and 2022 in six countries (China *n* = 16, Japan *n* = 9, Korea *n* = 6, USA *n* = 4, Argentina *n* = 2, and France *n* = 1). These studies focused on patients with critical illnesses, cancer, medical and surgical departments, and unselected hospitalized patients. The participants’ mean age ranged from 47 to 88 years old, and their BMI was from 20.7 to 28 kg/m^2^. The included studies have a mean mortality rate of 13% (ranging from 8.7 to 43.2%). The criteria and cut-offs for evaluating the sarcopenia varied among the studies. Twenty studies used regression analyses to investigate the relationship between CCR and mortality (5–7, 12, 14, 15, 18, 19, 23, 24, 31, 32, 37–40, 46, 49, 51). Regarding the relationship between CCR and muscle evaluation, 21 studies provided Pearson correlation coefficient (r) levels between CCR and CTASM, 12 evaluated the diagnostic value of CCR in sarcopenia, and six used adjusted HR/OR to predict sarcopenia.

**TABLE 1:** Characteristics of included studies in the current meta-analysis.

Study	Country	Design	Population	Sample	Age, year	Male, %	BMI	Follow-up	Mean CCR	Study quality
Abe et al. (45)	Japan	R, SC	Patients with CHF	248	77	49	22.4	In-hospital	NA	7
Barreto et al. (5)	USA	P, MC	ICU patients	171	63	61	26	90 days	84	9
Barreto et al. (13)	USA	R, SC	ICU patients	398	65	58	28	90 days	69	8
Chen et al. (31)	China	R, SC	Cancer	664	65	70	NA	28 months	76	8
Fujita et al. (21)	Japan	CS, SC	Patients with IPF	49	73	90	22.3	N/A	86	6
Fu et al. (34)	China	CS, SC	Cancer	182	55	63	21.6	N/A	79	8
Huang et al. (22)	China	CS, SC	AECOPD	104	67	100	20.6	N/A	96	6
Huang et al. (23)	China	R, SC	CAP	769	79	62	21.5	In-hospital	N/A	8
Lchikawa et al. (35)	Japan	R, SC	Chronic liver disease	303	66	50	23.8	NA	70	7
Jung et al. (14)	Korea	R, SC	ICU patients with RRT	1,588	65	60	25.4	90 days	92	8
Jung et al. (37)	Korea	R, SC	Cancer	3,060	61	54	23.6	1 year	82	8
Kashani et al. (15)	USA	R, SC	Lung transplant patients	28	58	54	25.9	1 year	106	6
Kashani et al. (12)	USA	R, SC	ICU patients	226	68	46	28	90 days	50	8
Kim et al. (19)	Korea	R, SC	Patients with CAD	1,928	65	71	24.9	3 years	110	8
Kim et al. (38)	Korea	R, SC	Patients with CABG	605	72	72	23.9	30 days PO	87	8
Lee et al. (39)	Korea	R, MC	Patients with CAD	1,086	72	63	24.6	3 years	105	8
Lchikawa et al. (36)	Japan	R, SC	Liver disease	313	65	48	23.6	NA	70	6
Lin et al. (41)	China	CS, SC	Non-d-CKD	272	66	57	26	N/A	100	8
Lin et al. (40)	China	R, SC	Non-d-CKD	1,141	71	58	25.5	Until death	98	8
Lin et al. (42)	China	CS, SC	CKD	297	69	57	26.3	N/A	51	8
Liu et al. (32)	China	R, SC	AIS	217	68	64	NA	3 months	71	8
Mauro et al. (43)	Argentina	R, SC	ALT	215	55	55	28.4	11.7 months	56	7
Nishiki et al. (20)	Japan	R, SC	COPD	201	72	95	22.3	NA	86	6
Osaka et al. (17)	Japan	P, MC	D2M	285	67	56	25.3	NA	NA	9
Okubo et al. (44)	Japan	R, SC	Patients with hip fracture	130	88	22	20.7	In-hospital	66	6
Ren et al. (6)	China	P, SC	Old patients	758	86	78	23	212 days	72	9
Romeo et al. (46)	Argentina	R, SC	Patients undergoing TAVR	100	84	36	27.2	1 year	69	7
Shin (47)	Korea	R, SC	D2M	1,577	63	58	25.2	NA	84	8
Sun et al. (18)	China	R, SC	Cancer	327	62	72	21.9	3 years	75	8
Tamai et al. (48)	Japan	R, SC	Cancer	50	65	60	24.1	36.5 months	79	6
Tang et al. (49)	China	P, SC	Cancer	579	59	65	23.2	Until death	73	9
Tang et al. (7)	China	R, SC	Hospitalized older patients	248	81	81	22.5	3 years	74	8
Ulmann et al. (50)	France	R, SC	Cancer	44	65	68	24.4	NA	79	6
Wang et al. (51)	China	R, SC	Neurocritically ill patients	538	57	63	22.9	In-NCU	70	8
Yang et al. (16)	China	R, SC	Postoperative cancer patients	417	58	60	23.3	30 days PO or in-hospital	61	6
Yang et al. (33)	China	R, SC	D2M	193	55	59	26.1	In-hospital	73	8
Yanishi et al. (52)	Japan	P, SC	Kidney transplant recipients	62	47	67	22.4	NA	104	7
Zheng et al. (24)	China	R, DB	Cancer	989	67	80	22.7	7 months	65	8

AECOPD, acute exacerbation of chronic obstructive pulmonary disease; AIS, acute ischemic stroke; R, retrospective; ALT, awaiting liver transplantation; BMI, Body Mass Index; CABG, coronary artery bypass grafting; CAD, coronary artery disease; CAP, community-acquired pneumonia; CCR, creatinine/cystatin C ratio; CHF, chronic heart failure; CKD, chronic kidney disease; COPD, chronic obstructive pulmonary disease; CS, cross-sectional; DB, data base; D2M, type 2 diabetes; ICU, intensive care unit; IPF, idiopathic pulmonary fibrosis; MC, multi-centers; NA, not available; N/A, not applicable; NCU, neurocritical care unit; P, prospective; PO, postoperative; RRT, renal replacement therapy; SC, single-center; TAVR, transcatheter aortic valve replacement.

The study quality ranged from moderate to high, according to the specifics of the quality evaluation in Supplementary Table 2 (Scores range from 6 to 9). Overall, 24 studies were judged to be of good quality, while 14 study was considered to be of moderate quality (Table 1).

### Findings from meta-analysis

#### Prediction of mortality by CCR

**FIGURE 2:**
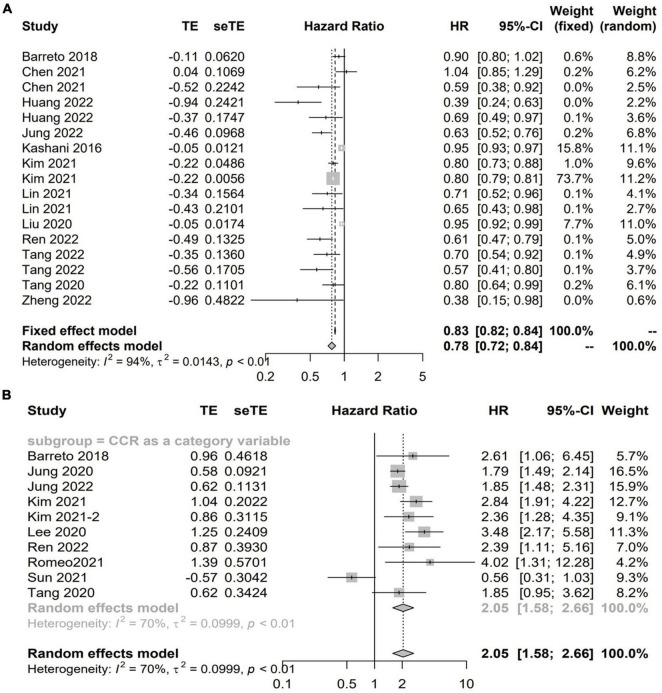
The forest plot in assessing the impact of creatinine/cystatin C ratio (CCR) on mortality in hospitalized patients by hazard ratio (HR) using regression analysis as continuous **(A)** and categorical variables **(B)**.

Twenty studies with 13,560 patients investigated the impact of CCR on mortality in hospitalized patients using HR (5–7, 12, 14, 15, 18, 19, 23, 24, 31, 32, 37–40, 46, 49, 51). Among these studies, 13 studies with 11,355 patients reported the CCR treated as a continuous variable, and the pooled results showed a higher CCR was independently associated with a lower risk of mortality (HR 0.78; 95% CI, 0.72–0.84; *I*^2^ = 94%; *P* < 0.01, Figure 2A; 5–7, 12, 19, 23, 24, 31, 32, 37, 38, 40, 49). A total of 10 studies including 9,164 patients reported the risk estimation according to CCR categories (6, 7, 13, 14, 18, 19, 37–39, 46). When pooled, there was a significant prognostic role for the CCR category on patients’ mortality (HR 2.05; 95% CI, 1.58–2.66; *I*^2^ = 93%; *P* < 0.0001). That is, patients with low values of CCR were less likely to survive than patients with high values (Figure 2B). A graph shaped symmetrical inverted funnel indicates there is no publication bias (Supplementary Figure 1).

Supplementary Figures 2–7 shows the detailed information of subgroup analyses by CCR categories and CCR treated as a continuous variable. Significant associations between CCR and all-cause mortality were also confirmed in most subgroups (Supplementary Figures 2–7).

#### The relationship between CCR and CTASM

**FIGURE 3:**
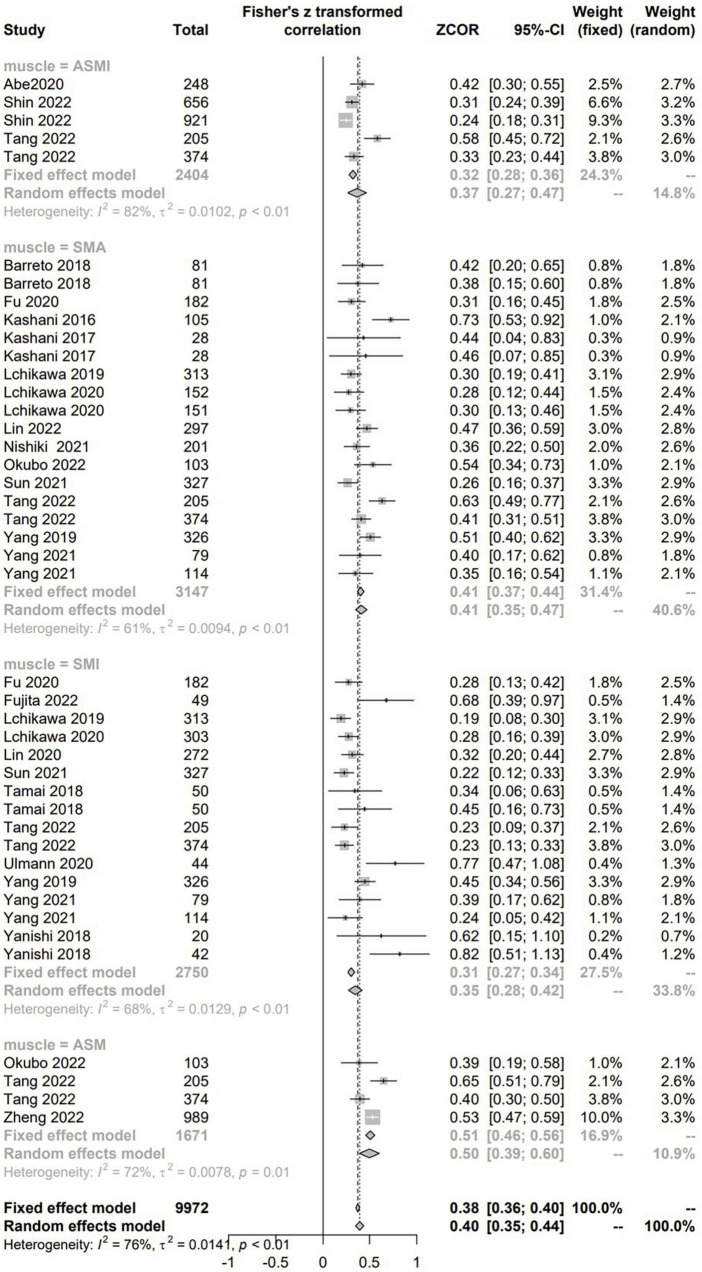
The pooled estimate of the relationship between serum creatinine/cystatin C ratio and computed tomography-assessed skeletal muscle.

There were 25 studies with 7,868 patients evaluated the correlation between CCR and CTASM from hospitalized patients using the correlation coefficient. As to the CTASM types, skeletal muscle area (SMA) was the most reported (*n* = 12), followed by the skeletal muscle index (SMI, defined as SMA divided by BSA, *n* = 11), appendicular skeletal muscle (*n* = 3), and appendicular skeletal muscle index (*n* = 3). The pooled results showed positive and significant correlations between CCR and all the four types of CTASM (Fisher’s Z ranged from 0.35 to 0.5; *P* values ranged from < 0.01 to 0.01) with the heterogeneity from 61 to 82% (Figure 3).

#### The diagnostic value of CCR for detecting sarcopenia

Twelve articles presented the AUC value for CCR in the diagnosis of sarcopenia (7, 21, 24, 34, 40–42, 45–47, 49, 50). Among them, two studies only reported AUC values [male/female: 0.813/0.613 (50); 0.752/0.754 (24)], and the other 10 provided the mean AUC and SE values. When pooled, the AUC value of CCR to predict sarcopenia was 0.689 (95% CI, 0.632–0.746; *I*^2^ = 82%; *P* < 0.01) (Supplementary Figure 8).

#### The relationship between CCR and HGS

**FIGURE 4:**
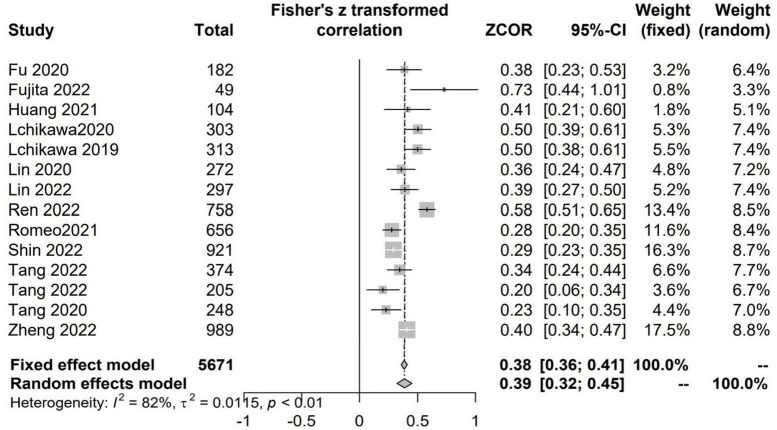
The pooled estimate of the relationship between serum creatinine/cystatin C ratio and handgrip strength.

There were 13 studies with 5,771 patients evaluated the correlation between CCR and HGS (6, 7, 21, 22, 24, 34–36, 41, 42, 46, 47, 49). There were positive and significant correlations between CCR and all the CTASM types (Fisher’s *Z* = 0.39; 95% CI, 0.32–0.45; *I*^2^ = 82%; *P* < 0.001) (Figure 4).

#### The relationship between CCR and GS

**FIGURE 5:**
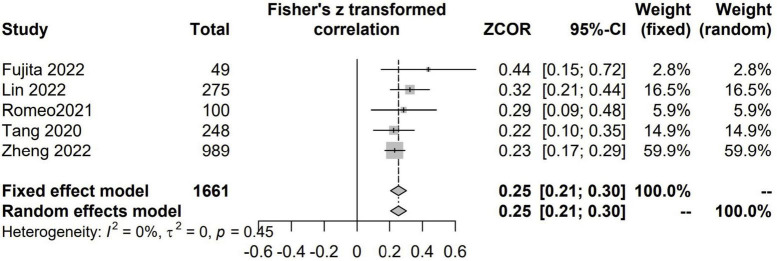
The pooled estimate of the relationship between serum creatinine/cystatin C ratio and gait speed.

There were 5 studies with 1,661 patients assessed the correlation between CCR and GS (7, 21, 24, 42, 46). There were positive and significant correlations between CCR and all the GS (Fisher’s Z = 0.25; 95% CI, 0.21–0.30; *I*^2^ = 0%; *P* < 0.001) (Figure 5).

#### The relationship between CCR and nutrition risk

Only four studies described the relationship between CCR and nutrition risk (6, 13, 16, 45). In the study by Abe et al. (45), the authors found that CCR was positively correlated with Mini Nutritional Assessment Short Form scores (*r* = 0.138, *P* = 0.03). Barreto et al. reported that CCR was a fair predictor of malnutrition as defined by the modified-NUTRIC score (AUC = 0.61) and with > 90% sensitivity and > 90% specificity (13). After adjusting for potential confounders, the CCR remained independently predictive of malnutrition [OR per 10-unit decrease in CCR, 1.15 (1.05, 1.26); *P* = 0.002]. Similar results were also seen in the study by Ren and coauthors, which showed that lower CCR was independently associated with nutritional risk/malnutrition whether or not CCR was treated as a continuous or category variable (6). In contrast, one study focused on cancer patients found no differences in the incidence of nutrition risk screening 2002 score ≥ 3 between the low and high CCR groups (*P* = 0.172) (16).

#### The relationship between CCR and other clinical outcomes

Three studies (12, 13, 51) provided data on the relationship between the CCR and the duration of MV, of which the study by Wang et al. suggested that CCR at admission significantly correlated with MV (*r* = 0.138, *P* = 0.001) (51). As to the other studies that provided specific data on this topic, one reported an increase in the CCR significantly predicted ventilator liberation [aHR 1.07 (0.97, 1.19); *P* = 0.18] (5), and the other suggested the duration of MV was significantly lower for those with a higher CCR [−1 day for each 10 units of CCR (95% CI, −1.4 to −0.2; *P* = 0.006)] (12).

Intensive care unit (ICU) and hospital LOS were available in three studies (5, 37, 51), of which the study by Wang et al. suggested that CCR at admission significantly correlated with ICU LOS (*r* = 0.161, *P* < 0.001) (51). Jung et al. found significant trends toward shorter ICU (*P* = 0.002) and hospital LOS (*P* < 0.001) in the higher CCR quartiles (37). Similarly, Barreto et al. suggested that an increase in the CCR significantly predicted more rapid discharge from the ICU [aHR, 1.06 (0.99, 1.14); *P* = 0.003] and hospital [aHR, 1.10 (1.03, 1.18) *P* = 0.007] (5).

Seven studies focused on the predictive value of CCR on the overall complications. The pooled findings from five studies that provided specific data on this topic showed that a decreased CCR was independently associated with a higher mortality risk (HR 1.66; 95% CI, 1.17–2.36; *I*^2^ = 83%; *P* < 0.01) (7, 16, 24, 38, 39; Supplementary Figure 9). In the remaining two studies, one reported that CCR was significantly lower in patients with cardiovascular disease (*P* = 0.008) and lower extremity arterial disease (*P* = 0.004) (33), and the other found no associations between CCR and adverse reactions (31).

## Discussion

We conducted the current meta-analysis of 38 studies to assess the value of CCR in hospitalized patients. Our results showed that CCR had a significant correlation with CTASM, GS and HGS, and the ROC curves suggested that CCR had good diagnostic efficacy for sarcopenia, indicating that CCR is an ideal alternative biomarker to screen sarcopenia. CCR, on the other hand, can reliably predict mortality in hospitalized patients, which was confirmed by regression analysis of CCR as both continuous and categorical variables. In addition, less evidence showed that higher CCR was independently associated with a shorten MV duration, reduced ICU and hospital LOS, less nutritional risk, and decreased complications in patients.

### CCR as a muscle mass evaluating tool in hospitalized patients

Recent findings suggest CCR is associated with disease-related catabolism because it reflects altered muscle mass (12). Serum creatinine is mainly produced by creatine phosphate during skeletal muscle metabolism (53). Therefore, patients with reduced muscle mass have lower creatinine levels. Also, cystatin C is a low molecular weight protein produced continuously by all nucleated cells and is readily filtered, absorbed, and broken down in the proximal renal tubules. It is not influenced by muscle metabolism (54). As a result, among patients with stable kidney function, one of the key factors of the difference between these two measures, skeletal muscle mass, is one of the primary determinants of the difference between these two markers. The effect of muscle mass on creatinine can be estimated by the ratio to cystatin C, which provides an easily accessible and reproducible assessment of muscle in patients at high risk for catabolism (12, 13).

Our results suggest that the CCR is an inexpensive, valid, objective, and reproducible tool for muscle mass assessment. We validated that CCR can screen for sarcopenia in three domains. CCR was significantly correlated not only with SAM/SMI, but also with GS and HGS. This supports the current sarcopenia definition in guidelines, that is, sarcopenia is assessed by skeletal muscle mass, muscle strength, and physical performance (55). Also, subgroup analyses suggested that medical, surgical, cancer, trauma, and ICU patients maintained consistently high correlations, meaning that CCR can be applied in various conditions (Figure 3).

In addition, the included studies used different definitions of sarcopenia. For example, most authors used diagnostic thresholds reported in previous studies to define sarcopenia or based on arbitrary thresholds from diverse populations (e.g., lowest 25th quartile, 33rd quartile, or median). These definitions were another source of heterogeneity in our results. Based on different sarcopenia definitions, we found that sarcopenia remained robustly correlated with CCR for all. On the other hand, different definitions have prevented obtaining a uniform CCR cut-off value for the diagnosis of sarcopenia in the current manuscript. Therefore, given the differences in disease, body size, dietary structure, and nutritional interventions among the included inpatients, there is still a need to establish appropriate CCR thresholds based on the local sarcopenic population in the future clinical application of CCR.

### CCR as a nutritional screening tool in hospitalized patients

Several studies included in our meta-analysis demonstrate that CCR can be utilized as a surrogate indication for a variety of nutritional screening techniques (6, 13, 16, 45). These studies found that CCR remained an independent predictor of nutrition risk/malnutrition in patients after adjusting for age, gender, Acute Physiology and Chronic Health Evaluation (APACHE) III score, Sequential Organ Failure Assessment (SOFA) score, and BMI (6, 13, 16, 45). Low CCR values generally indicate reduced muscle tissue, which is associated with malnutrition. In contrast, high CCR values indicate a complete muscle tissue mass and functional status and can identify malnutrition at the initial stage.

Furthermore, some studies suggest that CCR is superior to some traditional nutritional risk indicators (e.g., NUTRIC score), which are established and validated only for nutritional status and not as a surrogate for muscle mass (53). Thus, the independence from the subjective provision of information, weight data, and complex calculations, as well as the simplicity and repeatability, make CCR a favorable choice for clinical decision support of busy bedside clinicians compared to previous, more sophisticated tools. Although the studies we included involved a wide range of inpatient populations such as ICU, geriatric patients, and medical patients, given the number of studies, further confirmation in a larger sample is needed in the future.

### CCR as a prognostic indicator in hospitalized patients

We provide evidence for the association of sarcopenia with inpatient prognosis by evaluating CCR. A possible explanation for the association of CCR with mortality is that CCR may reflect muscle mass (12), the latter has been demonstrated to influence outcomes in various patient populations. Current literature suggests that sarcopenia is related to reduced protein synthesis and enhanced degradation induced by wasting, physical restraints, infection, prolonged mechanical ventilation and sedation, and muscle relaxants in various hospitalized settings (56). Especially in ICU patients with multiple organ dysfunction syndromes, the cross-sectional area of muscle fibers decreases at a rate of 3 to 4% per day, resulting in skeletal muscle atrophy, which affects vital physiological functions (4). As a result, muscle is also considered one of the failing organs of multiple organ dysfunction syndrome and has received widespread attention.

Although the pathophysiological relationship between muscle loss and patients’ prognosis is not fully understood, several studies have suggested it may be associated with a high catabolic state, cytokine impairment, and insulin signaling, leading to glucose intolerance (57). Sarcopenia also decreases the body’s ability to respond adequately to inflammatory stimuli and delays the implementation of rehabilitation (4, 57). Under these conditions, patient immune function may be reduced, leading to a high CCR, i.e., high risk of sarcopenia, associated with various complications, prolonged ICU or hospital stay, prolonged mechanical ventilation, and ultimately increased risk of patient death, as shown by our findings.

### Strengths and limitations of the study

To the best of our knowledge, this is the first meta-analysis to assess the value of CCR on muscle evaluation and prognosis in hospitalized patients. We included 38 studies with more than 20,000 patients with sufficient statistical power to conduct subgroup analysis based on potential influencing factors. The vast majority were based on multiple regression analyses of the included studies. Most of these included studies adjusted for a variety of possible confounders, including demographic variables (e.g., age and sex), anthropometric measures (e.g., BMI), nutritional status (e.g., subjective global assessment, NUTRIC score, disease severity (e.g., SOFA, APACHE-II score), disease-specific (e.g., tumor type, stage, and type of treatment), and physical fitness status as a prognostic factor. Thus, our findings somewhat control these confounding factors when evaluating muscle, prognosis, and other clinical outcome-related CCR measures.

Our meta-analysis has several limitations. (1) The observational design of all included studies excluded any causal inference. Also, patients included only in CCR tests in retrospective studies were prone to selection bias. (2) The small sample may be subject to false-positive bias; the small number of included studies in some subgroup analyses interpreted the results with caution. (3) The mean age of the included patients varied widely (47–88 years), but there was insufficient data to further explore the effect of age on mortality. (4) The uneven distribution of underlying disease in the study population may also have a different prognostic value. (5) CCR is unlikely to be at a steady state under AKI conditions, making the application of muscle mass ratios less desirable than in other clinical situations. Of note, cystatin C is a cathepsins inhibitor, and its levels increased in hyperthyroidism, obesity, metabolic syndrome, diabetes mellitus type 2, and different types of inflammation, albeit in different degrees (58, 59). However, most included studies did not exclude these confounding factors. (6) Patients’ medications or other interventions may affect the results of our study. Therefore, to reduce the effect of the intervention, we used the longest follow-up time of each included study. (7) The vast majority of the included studies were from Asian countries, so more data from other ethnicities are needed for confirmation.

## Conclusion

The results of our study indicated significant correlations between CCR and skeletal muscle evaluation and a prognostic role of CCR that higher circulating CCR levels were positively associated with the less risk of all-cause mortality in hospitalized patients. Thus, we recommended using CCR as a new prognostic biomarker to provide better information not only in decision correlations for muscle mass assessment but also in the prediction of survival and other associated clinical outcome.

## Data availability statement

The original contributions presented in this study are included in the article/Supplementary material, further inquiries can be directed to the corresponding author.

## Author contributions

W-HZ and YY contributed to analysis and drafting of the article. Y-BZ contributed to data collection and analysis. H-BH contributed to the conception of the study, design, revisions of this manuscript, and was responsible for the integrity of the work as a whole, from inception to publication of the article. All authors approved the final version submitted for publication.

## Footnotes

1
https://inplasy.com/inplasy-2022-9-0097/
