# Supplementary material for: Serum creatinine/cystatin C ratio as a muscle mass evaluating tool and prognostic indicator for hospitalized patients: A meta-analysis
Source: Front Med (Lausanne). 2023 Jan 9;9:1058464. doi: 10.3389/fmed.2022.1058464 (PMC9868859; doi:10.3389/fmed.2022.1058464)
Supplement: Supplementary file 1 [file Data_Sheet_1.docx]

**Serum** **creatinine/cystatin C ratio as a muscle mass evaluating tool and prognostic indicator for hospitalized patients: a meta-analysis**

**Supplementary material**

**Additional file 1**

Checklist following PRISMA guideline………………………………………………………………………………………………….……..1

**Additional file 2**

Objectives, predefined outcomes, and main results of included studies……………………………………………...……..5

**Additional file 3**

Table S2: Quality assessment and overall risk of bias of included studies………………….…………………..….…......11

**Additional file 4**

Figure S1: Publication bias …….………………………………………………………….…...………………….……….…….……......…13

**Additional file 5**

Figure S2. The forest plot in assessing the impact of CCR on mortality by HR using regression analysis as continuous variables by subgroup of patient population................................................................................................ 14

Figure S3. The forest plot in assessing the impact of CCR on mortality by HR using regression analysis as continuous variables by subgroup of geographic location….……………........................................................................…15

Figure S4. The forest plot in assessing the impact of CCR on mortality by HR using regression analysis as continuous variables by subgroup of gender……………………………………………………………………………………...........…16

Figure S5. The forest plot in assessing the impact of CCR on mortality by HR using regression analysis as categorical variables by subgroup of population…………………………………………………………………………...…...........…17

Figure S6. The forest plot in assessing the impact of CCR on mortality by HR using regression analysis as categorical variables by subgroup of geography location.….……………………………………………………………............…18

Figure S7. The forest plot in assessing the impact of CCR on mortality by HR using regression analysis as categorical variables by subgroup of gender.…………………………………………………………………………...………...........…19

**Additional file 6**

Figure S8: The pooled estimate of the diagnostic value of CCR for detecting sarcopenia…………...…...….……20

**Additional file 7**

Figure S9: The pooled estimate of the relationship between serum creatinine/cystatin C ratio and complications….…………………………………………………………………………………………………………………………………………...21

**Additional file 1**

**PRISMA checklist**

| **Section/topic** | **#** | **Checklist item** | **Reported on page #** |
| --- | --- | --- | --- |
| **TITLE** | | |  |
| Title | 1 | Identify the report as a systematic review, meta-analysis, or both. | 1 |
| **ABSTRACT** | | |  |
| Structured summary | 2 | Provide a structured summary including, as applicable: background; objectives; data sources; study eligibility criteria, participants, and interventions; study appraisal and synthesis methods; results; limitations; conclusions and implications of key findings; systematic review registration number. | 2 |
| **INTRODUCTION** | | |  |
| Rationale | 3 | Describe the rationale for the review in the context of what is already known. | 4 |
| Objectives | 4 | Provide an explicit statement of questions being addressed with reference to participants, interventions, comparisons, outcomes, and study design (PICOS). | 5 |
| **METHODS** | | |  |
| Protocol and registration | 5 | Indicate if a review protocol exists, if and where it can be accessed (e.g., Web address), and, if available, provide registration information including registration number. | 6 |
| Eligibility criteria | 6 | Specify study characteristics (e.g., PICOS, length of follow-up) and report characteristics (e.g., years considered, language, publication status) used as criteria for eligibility, giving rationale. | 6 |
| Information sources | 7 | Describe all information sources (e.g., databases with dates of coverage, contact with study authors to identify additional studies) in the search and date last searched. | 6 |
| Search | 8 | Present full electronic search strategy for at least one database, including any limits used, such that it could be repeated. | 6 and Additional file 2 |
| Study selection | 9 | State the process for selecting studies (i.e., screening, eligibility, included in systematic review, and, if applicable, included in the meta-analysis). | 6-7 |
| Data collection process | 10 | Describe method of data extraction from reports (e.g., piloted forms, independently, in duplicate) and any processes for obtaining and confirming data from investigators. | 7 |
| Data items | 11 | List and define all variables for which data were sought (e.g., PICOS, funding sources) and any assumptions and simplifications made. | 7 |
| Risk of bias in individual studies | 12 | Describe methods used for assessing risk of bias of individual studies (including specification of whether this was done at the study or outcome level), and how this information is to be used in any data synthesis. | 7 |
| Summary measures | 13 | State the principal summary measures (e.g., risk ratio, difference in means). | 7 |
| Synthesis of results | 14 | Describe the methods of handling data and combining results of studies, if done, including measures of consistency (e.g., I^2^) for each meta-analysis. | 7-8 |

| Risk of bias across studies | 15 | Specify any assessment of risk of bias that may affect the cumulative evidence (e.g., publication bias, selective reporting within studies). | 8 |
| --- | --- | --- | --- |
| Additional analyses | 16 | Describe methods of additional analyses (e.g., sensitivity or subgroup analyses, meta-regression), if done, indicating which were pre-specified. | 8-9 |
| **RESULTS** | | |  |
| Study selection | 17 | Give numbers of studies screened, assessed for eligibility, and included in the review, with reasons for exclusions at each stage, ideally with a flow diagram. | 10  Figure 1 |
| Study characteristics | 18 | For each study, present characteristics for which data were extracted (e.g., study size, PICOS, follow-up period) and provide the citations. | 10  Table 1 |
| Risk of bias within studies | 19 | Present data on risk of bias of each study and, if available, any outcome level assessment (see item 12). | 10-11 |
| Results of individual studies | 20 | For all outcomes considered (benefits or harms), present, for each study: (a) simple summary data for each intervention group (b) effect estimates and confidence intervals, ideally with a forest plot. | 11-12 |
| Synthesis of results | 21 | Present results of each meta-analysis done, including confidence intervals and measures of consistency. | 11-14 Additional file 3-5 |
| Risk of bias across studies | 22 | Present results of any assessment of risk of bias across studies (see Item 15). | 10-11 Additional file 2 |
| Additional analysis | 23 | Give results of additional analyses, if done (e.g., sensitivity or subgroup analyses, meta-regression [see Item 16]). | 11-14 |
| **DISCUSSION** | | |  |
| Summary of evidence | 24 | Summarize the main findings including the strength of evidence for each main outcome; consider their relevance to key groups (e.g., healthcare providers, users, and policy makers). | 15 |
| Limitations | 25 | Discuss limitations at study and outcome level (e.g., risk of bias), and at review-level (e.g., incomplete retrieval of identified research, reporting bias). | 19-20 |
| Conclusions | 26 | Provide a general interpretation of the results in the context of other evidence, and implications for future research. | 21 |
| **FUNDING** | | |  |
| Funding | 27 | Describe sources of funding for the systematic review and other support (e.g., supply of data); role of funders for the systematic review. | 23 |

**Additional file 2**

**Objectives, predefined outcomes, and main results of included studies**

| Study | Objective | Outcome | Main results |
| --- | --- | --- | --- |
| Abe 2020^[45]^ | To examine whether CCR is a better and more accurate index of muscle mass in patients with chronic heart failure. | ③⑦ | ASM were positively correlated with CCR (r =0.399). CCR had a low diagnostic value of MW (ROC curve AUC=0.52). In addition, MNASF scores were positively correlated with CCR (r=0.138, P=0.030) |
| Barreto 2018^[5]^ | To assess the validity of the CCR as a predictor of muscle mass. | ③ | The CCR correlated with CTMSA (r = 0.40). After adjustment for age, sex, severity of illness, and BMI, CCR was independently associated with muscle mass (P = 0.001). A decrease in the CCR (indicative of lower muscle mass) was also associated with frailty and worse short-term clinical outcomes. |
| Barreto 2018^[13]^ | Using CCR in identifying malnutrition in the ICU and compared it with the modified-NUTRIC score. | ①②③ | The CCR was significantly lower in malnourished patients than in well-nourished patients (64 ± 27 vs 72 ± 25; P = 0.002), and reductions in CCR corresponded to increased malnutrition severity (P = 0.001). As a screening tool, the CCR was an indicator of malnutrition risk (AUC 0.61). Patients with a low CCR had a significantly higher risk of mortality (HR = 2.61, 95% CI 1.06–6.48, P = 0.038). |
| Chen2021^[31]^ | To assess the predictability value of CCR in determining potential chemotherapy-induced undesirable reactions and eventual death of older patients diagnosed with NSCLC. | ①⑥ | After adjusting for confounding factors, there was no association between the CCR and adverse reactions. High CCR was independently associated with a lower risk of mortality after adjusting for confounding factors in females (HR=0.593,95% CI: 0.382-0.92; p=0.02) but not in males. |
| Fujita 2022^[21]^ | To confirm the utility of the CCR in predicting sarcopenia and investigate its clinical relevance. | ②④⑤⑦ | CCR had a good diagnostic value of MW (ROC curve AUC=0.85). Sarcopenia was identified more in patients with a low serum CCR (P<0.001). In addition, CCR was an independent predictive marker of worse PROs evaluated using mMRC (P<0.05), CAT (P<0.05), and K-BILD (P<0.05). |
| Fu 2020^[34]^ | To assess whether CCR is associated with sarcopenia and cancer-related fatigue in advanced cancer patients. | ③④⑦ | The CCR was significantly lower in both the sarcopenia and severe fatigue groups. We found significant associations between the CCR and SMA (r = 0.299; P <0.001), SMI (r = 0.269; P < 0.001) and HGS (r = 0.364; P < 0.001). |
| Huang 2021^[22]^ | To explored the value of CCR in diagnosing the reduction of muscle strength in men with AECOPD. | ④⑦ | Among AECOPD patients, CCR was lower in the low muscle strength group than in the normal muscle strength group. The CCR was correlated with HGS. The AUC for low handgrip strength was greater for the CCR than for Cr. CCR <0.99 was a risk factor for decreased muscle strength in male patients with AECOPD. |
| Huang 2022^[23]^ | To assess the association between CCR and septic shock risk and mortality in older patients with CAP. |  | Following adjustment for confounders, there was a significant association between high CCR and a lower risk of septic shock in female patients (OR=0.38, 95%CI: 0.16–0.94; p<0.05). After adjustment for confounding factors and irrespective of sex, high CCR was a protective factor for mortality in older adults with CAP (total group: HR=0.64, 95%CI: 0.48–0.84; p<0.05; male: HR=0.69, 95%CI: 0.49–0.97; p<0.05; female: HR=0.39, 95%CI: 0.24–0.62; p<0.05). |
| Lchikawa2020^[35]^ | The applicability of CCR for liver disease were evaluated. | ③④ | In the 313 patients, CCR was correlated with BMI and HGS. In patients evaluated with CT, the correlation coefficient for CCR with SM was 0.293. |
| Jung 2021^[14]^ | To evaluate the association of CCR with outcomes in patients with AKI undergoing CKRT. | ② | The 30- and 90-day mortality rates were significantly lower for the higher CCR groups. Multivariable Cox proportional hazards regression analyses revealed that the mortality risk decreased successively across quartiles of increasing CCR. These associations remained significant even after adjustment for confounding variables. |
| Jung 2022^[37]^ | To assess the association between CCR and survival in cancer patients. | ①② | In the Cox proportional hazards analysis, an increase in the CCR was associated with a significant decrease in the 6-month mortality (per 1 CCR, hazard ratio [HR] 0.35; 95% confidence interval [CI], 0.28–0.44).Analysis of 1-year mortality outcomes revealed similar findings. These associations were independent of confounding factors. |
| Kashani 2017^[15]^ | Sought to validate CCR among lung transplant patients. |  | Correlation coefficient between CCR and SM-CSA was significant at L2 (0.43; P=.02 and L3 (0.41; P=.03). |
| Kashani 2016^[12]^ | To evaluate CCR and its association with muscle mass and patient outcomes. | 1. ③ | The correlation (r) between CCR and muscle mass was 0.62 and coefficient of determination (r^2^) was 0.27 (P<0.0001). After adjustment for APACHE III, BMI, and age, CCR was independently predictive of both hospital (p = 0.001) and 90-day mortality (P< 0.0001). |
| Kim 2021^[19]^ | To assess CCR as a measured surrogate marker of low muscle mass. | ①② | The risk of 3-year mortality increased proportionally according to the decrease of CCR. CCR-based low muscle mass groups showed significantly higher risk of death, after adjusting for possible confounders. Low CCR values were associated with high mortality rate in patients who were ≥65 years, nonobese, male, had renal dysfunction at baseline, and presented with acute myocardial infarction. |
| Kim 2021-2^[38]^ | Aimed to investigate the associations between CCR and postoperative pulmonary complications in elderly patients undergoing of-pump CABG. | ②⑥ | A 10-unit increase of CCR was associated with a reduced risk of postoperative pulmonary complications (OR: 0.80, 95% CI: 0.69–0.92, P= 0.001). A low CCR was associated with an increased risk of developing postoperative pulmonary complications after off-pump CABG. |
| Lee 2020^[39]^ | To demonstrate the association of the CCR with clinical outcomes including cardiovascular and bleeding risk in older patients who underwent PCI. | ②⑥ | The lowest CCR quartile group (Q1) had a significantly higher 3-year MACE rate (Q1 vs. Q2–4; 23.1% vs. 11.2%, P< 0.001), while bleeding event rates were similar between the groups (Q1 vs. Q2–4; 2.6% vs. 2.2%, P= 0.656). The Cox proportional hazard model showed that lower CCR is an independent predictor for MACE events (HR 2.23, 95% CI 1.62–3.07, P< 0.001). |
| Lchikawa 2019^[36]^ | We evaluated the applicability of using CCR as screening method for sarcopenia. | ③ | SM was positively related to BW and weakly positive to CCR (R: M=0.287; F=0.271) |
| Lin 2020^[41]^ | To evaluate the feasibility of CCR in the assessment of muscle wasting. | ③④⑦ | The CCR was significantly lower in both the low SMI and low HGS groups. Moreover, the CCR correlated with SMI (r= 0.306, P< 0.001) and HGS (r =0.341, P<0.001). For the prediction of low SMI, CCR had an acceptable power in male patients (AUC =0.710, 95% CI =0.631-780), but not in female patients (AUC=0.533, 95% CI =.438-626). |
| Lin2021^[40]^ | To evaluate the association between CCR and mortality in patients with CKD. | ①⑦ | Compared to the survivor group, CCR, was lower in the non-survivors (P< 0.001). The AUC curve of serum CCR for predicting mortality had significant discriminative power. Based on the Cox proportional hazard models, lower values of CCR, both as continuous and categorical variables, independently predicted mortality. |
| Lin 2022^[42]^ | to evaluate the diagnostic validity of the novel CCR in advanced CKD. | ③④⑤⑦ | The CCR correlated moderately with SMM (r = 0.503, P < 0.001), HGS (r = 0.508, P < 0.001), and gait speed (r = 0.381, P < 0.001). For sarcopenia prediction, the CCR had acceptable discriminative powers in males [AUC=0.646, 95% CI 0.569–0.718] and females (AUC=0.754, 95% CI 0.670–0.826). |
| Liu 2020^[32]^ | To explore the predictive value of serum CCR in acute ischemic stroke patients receiving nutritional intervention. | ① | In multivariable logistic regression analyses, CCR at admission was independently associated with 3-month poor outcomes (OR: 0.953, 95% CI: 0.921-0.986, P=0.006) and 30-day mortality (OR: 0.953, 95% CI: 0.921-0.986, P= 0.006). |
| Mauro 2021^[43]^ | To evaluate the clinical factors associated with the presence of sarcopenia by SMI | ⑦ | CCR was the independent factors associated with the presence of sarcopenia (OR: 0.03, p = 0.007). |
| Nishiki 2021^[20]^ | To assess whether the CCR can be used as a predictive marker of pulmonary function and disease severity in patients with COPD. | ③ | There was a significant correlation between the ESM-CSA and the CCR. The CCR was significantly associated with forced vital capacity and forced expiratory volume in 1 second values, especially in former smokers. |
| Osaka 2018^[17]^ | We hypothesized that the CCR could be used as a marker for sarcopenia. | ③⑦ | The CCR was associated with an increased risk of sarcopenia [OR per 0.01 increment, 1.05; 95% CI, 1.01–1.09] after adjusting for covariates. AUC curve analysis indicated that the optimal the CCR cut-off point for identifying sarcopenia was 0.9, with an AUC, sensitivity, and specificity of 0.683 (95% CI, 0.573–0.793), 0.80, and 0.48, respectively. |
| Okubo 2022^[44]^ | To test whether the CCR can predict preoperative and early postoperative functional outcomes. | ③ | The correlations of CCR with CT values and CSA were as follows: iliopsoas at the apex of the femoral head, r = 0.40, P< 0.001 and r = 0.49, P<0.001, respectively; rectus femoris at the level of the lessor trochanter, r = 0.26, P = 0.007 and r = 0.37, P< 0.001, respectively. ROC analysis for predicting postoperative walking ability in preoperative independent patients with hip fracture and o femoral neck fractures AUC (95% CI ) of 0.63 (0.50–0.76) and 0.80 (0.65–0.96), respectively. |
| Ren 2022^[6]^ | To investigate the association of CCR with mortality, nutritional risk/malnutrition and sarcopenia among hospitalized older adults. | ①②④ | A high CCR (per 1-SD was 22.1) was independently associated with all-cause mortality (HR per 1-SD = 0.61, 95% CI: 0.47–0.79), nutritional risk/malnutrition (OR per 1-SD = 0.38, 95% CI: 0.29-0.49) and sarcopenia (OR per 1-SD = 0.58, 95% CI: 0.45–0.74). High CCR was positively correlated with hand grip strength (HGS) (r = 0.52, P < 0.001). |
| Romeo2021^[46]^ | To determine the predictive value of CCR as a surrogate marker of sarcopenia, and investigate its association with clinical outcomes after TAVR. | ②④⑤⑦ | CCR was significantly correlated with Gait Speed (r = 0.278, P=0.005). Moreover, survival curves were significantly worse (Log-rank test = P=0.02) and CHF readmissions were more prevalent in the lowest CCR (Log-rank test = P=0.01). In multivariate Cox regression analysis, we identified low CCR (cutoff ≤66) as an independent predictor of long-term adverse outcomes (HR = 4.01, 95% CI = 1.31–12.27, P=0.015) at 1-year follow-up. |
| Shin 2022^[47]^ | We investigated the associations of CCR with sarcopenia and carotid plaque score in patients with type 2 diabetes mellitus. | ③④ | Positive correlations between CCR and ASM/BMI (r = 0.239 in men and 0.303 in women, P< 0.001) and GS (r= 0.282 in men and 0.270 in women, P< 0.001) were observed in both genders. |
| Sun 2022^[18]^ | To investigate the validity of CCR as a predictor of sarcopenia and assess the relationship between CCR and prognosis of patients with gastric cancer. | ②③⑦ | CCR was significantly correlated with SMI (r=0.221, P<0.001) and SMA (r=0.258, P<0.001). The area under the curve for sarcopenia was significantly larger for CCR than for other biomarkers (CCR: 0.644, CysC: 0.535, Cr: 0.556). Patients in the high CCR group have longer survival time than that in low-CCR group, defined by the cut-off value 0.67. The C-index of both CCR and SMI with OS was 0.63. |
| Tamai 2018^[48]^ | To investigate whether the ratio between estimated eGFRcre/eGFRcys can be used as a predictive marker of survival in HCC patients. | ③⑦ | CCR was correlated with skeletal muscle mass index (r=0.331, P=0.019) and psoas muscle area index (r=0.397, P=0.004) in chronic liver disease patients. |
| Tang 2022^[49]^ | To explore and validate the diagnostic values of the CCR for determining sarcopenia in non-small cell lung cancer and to explore their prognostic values for overall survival. | ①③④⑦ | Both CCR and SI positively correlated with muscle mass and HGS. The optimal cutoff value for CCR was 0.623 in men and 0.600 in women, with AUC of 0.837 [95% CI: 0.770–0.904] in men (P = 0.25), and 0.808 (95% CI: 0.682–0.935) in women (P = 0.11), respectively. CCR-defined sarcopenia was independently associated with a high mortality risk [HR=.75, 95% CI: 1.25–2.44]. |
| Tang 2020^[7]^ | To investigate the association of the CCR with 3-year mortality and readmission among older inpatients | ①②④⑤⑥ | The CCR was positively correlated with BMI (r=0.214, p=0.001), calf circumference (r=0.253, p<0.001), HGS (r=0.244, p<0.001), and gait speed (r=0.221, p<0.001). A higher CCR was independently associated with a lower risk of 3-year all-cause mortality after adjusting for potential confounders (HR per 1-SD=0.80, 95% CI: 0.63–0.97). The CCR was not significantly associated with readmission (HR per 1-SD=0.97, 95% CI: 0.77-1.25). |
| Ulmann 2020^[50]^ | To evaluate CCR ability to detect myopenia in cancer patients compared to the CT-scan use as a standard. | ③ | CCR was well correlated with CT-scan Lean body mass (r=0.648 respectively). In terms of ability to detect myopenia, AUC for CCR were 0.813 and 0.673 8 for men and women, respectively. |
| Wang 2019^[51]^ | To explore the predictive value of CCR in neurocritically ill patients. | ①⑥ | CCR was significantly correlated with BMI (r=0.161, P< .001). In multivariate logistic regression analysis, we identified CCR as an independent pre‐dictor of long‐term functional outcome (OR: 0.989, 95% CI: 0.980–0.998, P=0.015) but not 30‐day mortality (P=0.513). |
| Yang 2019^[16]^ | To describe SI-based glomerular filtration rate, and investigate its association with short-term complications after curative resection of colorectal cancer. | ③⑥ | CCR showed a stronger correlation with SMA (r = 0.469, P< 0.001). |
| Yang 2021^[33]^ | We investigated the predictive value of CCR for the skeletal muscle composition and its correlations with glucose disposal ability and diabetic complications in patients with type 2 diabetes. | ③⑥ | CCR was significantly correlated with both the SMI (r=0.375, p<0.001) and MMA (r=0.378, P<0.001). In the diabetic complications analysis, CCR was significantly lower in patients with cardiovascular disease (95% CI (−1.47 to –0.22), p=0.008) and lower extremity arterial disease (95% CI (−1.44 to –0.29), P=0.004). |
| Yanishi 2018^[52]^ | To explore a more practical and simple method in detecting sarcopenia. | ③ | There was a significant positive correlation between the SMI and CCR in the male (r=0.553; p<0.001) and female groups (r=0.675; p<0.001). |
| Zheng 2022^[24]^ | To employ the simple and economic indicator CCR to screen for sarcopenia and to evaluate its prognostic value in patients with esophageal cancer | ①③④⑤⑥ | CCR showed the diagnostic value of sarcopenia. Individuals with CCR ≤ 68 had a poorer overall survival (HR=2.14, 95% CI: 1.71–2.68, P<0.001), and the all-cause mortality risk gradually decreased with the increase in CCR. There was a linear correlation between CCR and SMI. Patients in the high sarcopenia risk group showed a higher incidence of complications (OR=3.50, 95% CI: 1.85-6.61, P<0.001) and poorer long-term survival (HR=2.62, 95% CI: 1.02-6.77, P=0.046). |

① Impact of serum creatinine/cystatin C ratio on mortality in hospitalized patients by HR using regression analysis as continuous variables

② Impact of serum creatinine/cystatin C ratio on mortality in hospitalized patients by HR using regression analysis as categorical variables

③ Relationship between serum creatinine/cystatin C ratio and computed tomography-assessed skeletal muscle

④ Relationship between serum creatinine/cystatin C ratio and handgrip strength

⑤ Relationship between serum creatinine/cystatin C ratio and gait speed

⑥ Relationship between serum creatinine/cystatin C ratio and complications

⑦ estimate of the diagnostic value of serum creatinine/cystatin C ratio for detecting sarcopenia

AECOPD=acute exacerbation of chronic obstructive pulmonary disease; AKI=acute kidney injury; BMI=body surface area; CABG=coronary artery bypass surgery; CAT=chronic obstructive pulmonary disease assessment test; CCR=creatinine/cystatin ratio C; CKRT=continuous kidney replacement therapy; CSA=cross -sectional surface area; CKD=chronic kidney disease; K-BILD=King’s Brief Interstitial Lung Disease; MACE=major adverse cardiovascular events; MRC= medical research council; MNASF=Mini Nutritional Assessment Short Form scores; NSCLC=non-small cell lung cancer; SMA=skeletal muscle area; SMM=skeletal muscle mass; PCI=percutaneous coronary intervention; TAVR=transcatheter aortic valve replacement.

**Additional file 2**

**Quality assessment and overall risk of bias of included studies**

| First author / year | Patient selection | | | | Comparability | Outcome | | | Risk of bias |
| --- | --- | --- | --- | --- | --- | --- | --- | --- | --- |
|  | Representation of the exposed cohort | Selection of the non-exposed cohort | Ascertainment of exposure | Outcome of  interest not  present at start | Comparability of cohorts on the basis of the design or analysis | Assessment  of outcome | Was follow-up long enough for outcomes to occur | Adequacy of follow up of cohorts |  |
| Abe2020[45] | ★ | ★ | ★ | ☆ | ★☆ | ★ | ★ | ★ | 7 |
| Barreto 2018[5] | ★ | ★ | ★ | ★ | ★★ | ★ | ★ | ★ | 9 |
| Barreto 2018[13] | ★ | ★ | ★ | ☆ | ★★ | ★ | ★ | ★ | 8 |
| Chen2021[31] | ★ | ★ | ★ | ☆ | ★★ | ★ | ★ | ★ | 8 |
| Fujita 2022[21] | ★ | ★ | ★ | ☆ | ☆☆ | ★ | ★ | ★ | 6 |
| Fu 2020[34] | ★ | ★ | ★ | ☆ | ★★ | ★ | ★ | ★ | 8 |
| Huang 2021[22] | ★ | ★ | ★ | ☆ | ☆☆ | ★ | ★ | ★ | 6 |
| Huang 2022[23] | ★ | ★ | ★ | ☆ | ★★ | ★ | ★ | ★ | 8 |
| Lchikawa2020[35] | ★ | ★ | ★ | ★ | ☆☆ | ★ | ★ | ★ | 7 |
| Jung 2021[14] | ★ | ★ | ★ | ☆ | ★★ | ★ | ★ | ★ | 8 |
| Jung 2022[37] | ★ | ★ | ★ | ☆ | ★★ | ★ | ★ | ★ | 8 |
| Kashani 2017[15] | ★ | ★ | ★ | ☆ | ☆☆ | ★ | ★ | ★ | 6 |
| Kashani 2016[12] | ★ | ★ | ★ | ☆ | ★★ | ★ | ★ | ★ | 8 |
| Kim 2021[19] | ★ | ★ | ★ | ☆ | ★★ | ★ | ★ | ★ | 8 |
| Kim 2021-2[38] | ★ | ★ | ★ | ☆ | ★★ | ★ | ★ | ★ | 8 |
| Lee 2020[39] | ★ | ★ | ★ | ☆ | ★★ | ★ | ★ | ★ | 8 |
| Lchikawa 2019[36] | ★ | ★ | ★ | ☆ | ☆☆ | ★ | ★ | ★ | 6 |
| Lin 2020[41] | ★ | ★ | ★ | ☆ | ★★ | ★ | ★ | ★ | 8 |
| Lin2021[40] | ★ | ★ | ★ | ☆ | ★★ | ★ | ★ | ★ | 8 |
| Lin 2022[42] | ★ | ★ | ★ | ☆ | ★★ | ★ | ★ | ★ | 8 |
| Liu 2020[32] | ★ | ★ | ★ | ☆ | ★★ | ★ | ★ | ★ | 8 |
| Mauro 2021[43] | ★ | ★ | ★ | ☆ | ☆★ | ★ | ★ | ★ | 7 |
| Nishiki 2021[20] | ★ | ★ | ★ | ☆ | ☆☆ | ★ | ★ | ★ | 6 |
| Osaka 2018[17] | ★ | ★ | ★ | ★ | ★★ | ★ | ★ | ★ | 9 |
| Okubo 2022[44] | ★ | ★ | ★ | ☆ | ☆☆ | ★ | ★ | ★ | 6 |
| Ren 2022[6] | ★ | ★ | ★ | ★ | ★★ | ★ | ★ | ★ | 9 |
| Romeo2021[46] | ★ | ★ | ★ | ☆ | ☆★ | ★ | ★ | ★ | 7 |
| Shin 2022[47] | ★ | ★ | ★ | ☆ | ★★ | ★ | ★ | ★ | 8 |
| Sun 2022[18] | ★ | ★ | ★ | ☆ | ★★ | ★ | ★ | ★ | 8 |
| Tamai 2018[48] | ★ | ★ | ★ | ☆ | ☆☆ | ★ | ★ | ★ | 6 |
| Tang 2022[49] | ★ | ★ | ★ | ★ | ★★ | ★ | ★ | ★ | 9 |
| Tang 2020[7] | ★ | ★ | ★ | ☆ | ★★ | ★ | ★ | ★ | 8 |
| Ulmann 2020[50] | ★ | ★ | ★ | ☆ | ☆☆ | ★ | ★ | ★ | 6 |
| Wang 2019[51] | ★ | ★ | ★ | ☆ | ★★ | ★ | ★ | ★ | 8 |
| Yang 2019[16] | ★ | ★ | ★ | ☆ | ☆☆ | ★ | ★ | ★ | 6 |
| Yang 2021[33] | ★ | ★ | ★ | ☆ | ★★ | ★ | ★ | ★ | 8 |
| Yanishi 2018[52] | ★ | ★ | ★ | ★ | ☆☆ | ★ | ★ | ★ | 7 |
| Zheng 2022[24] | ★ | ★ | ★ | ☆ | ★★ | ★ | ★ | ★ | 8 |

**Additional file 3**


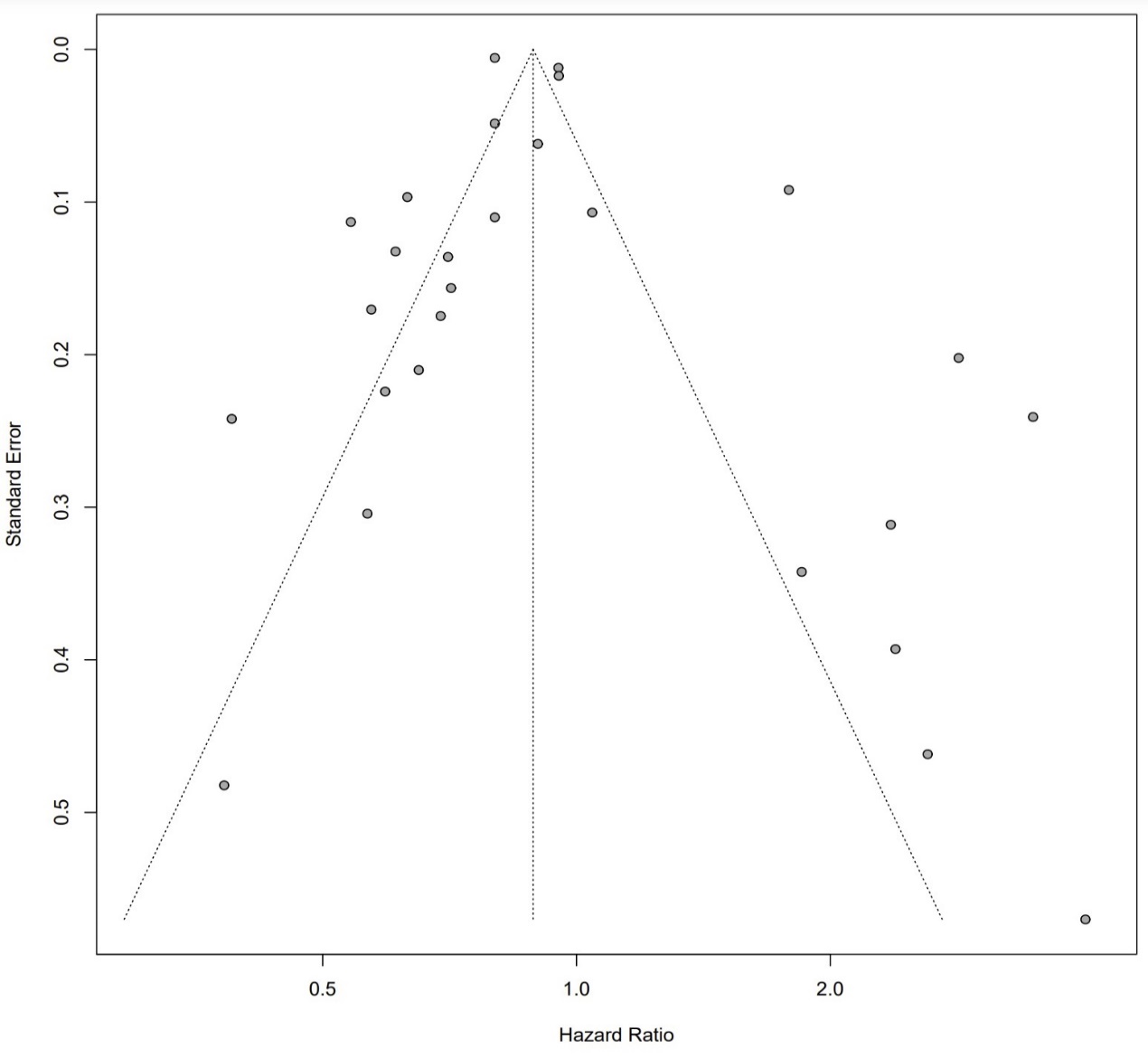


**Fig S1. The pooled estimate of the publication bias (Outcomes: prediction of mortality by CCR)**

**Additional file 4**


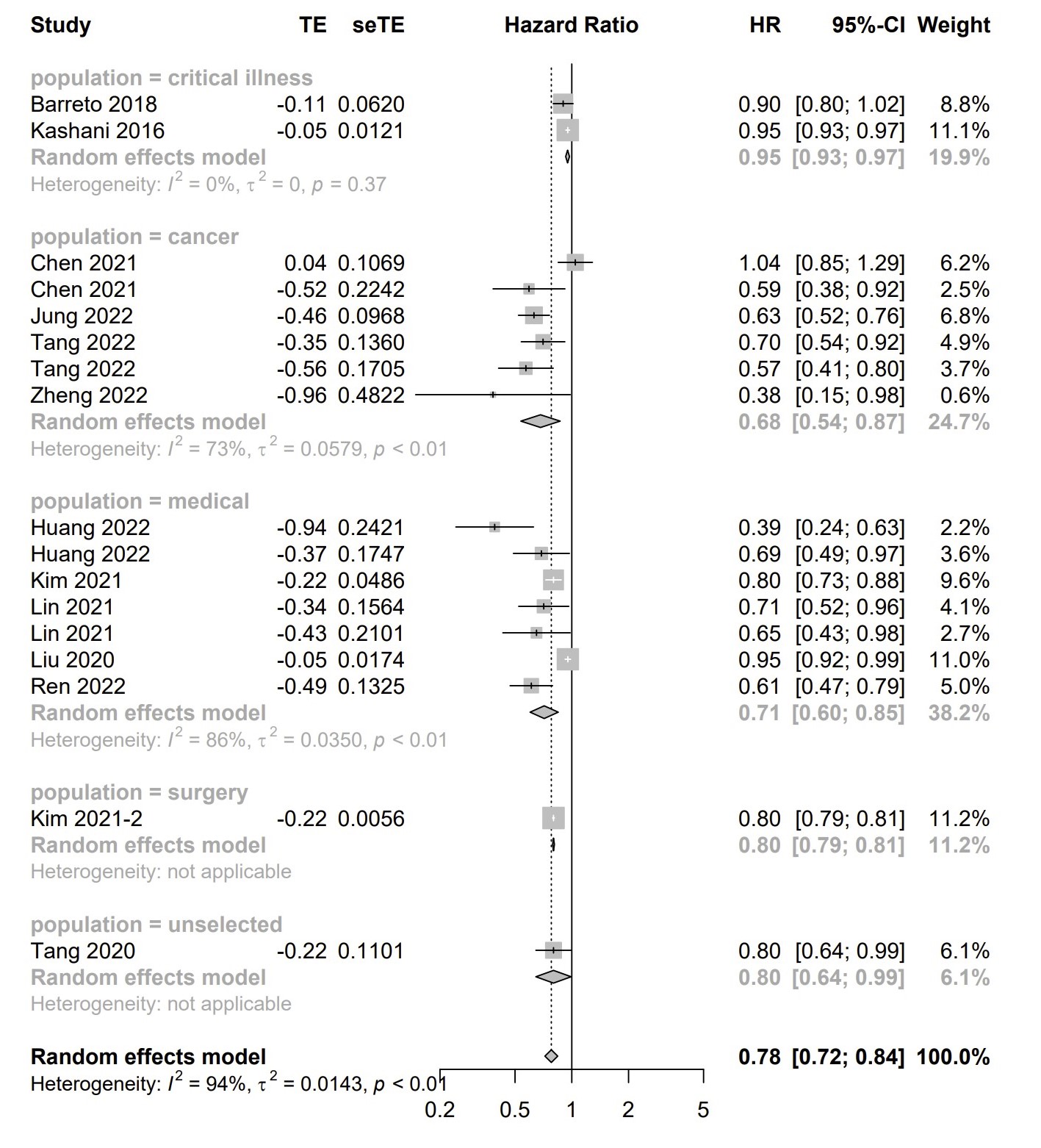


**Fig S2. The forest plot in assessing the impact of CCR on mortality by HR using regression analysis as continuous variables by subgroup of patient population.**


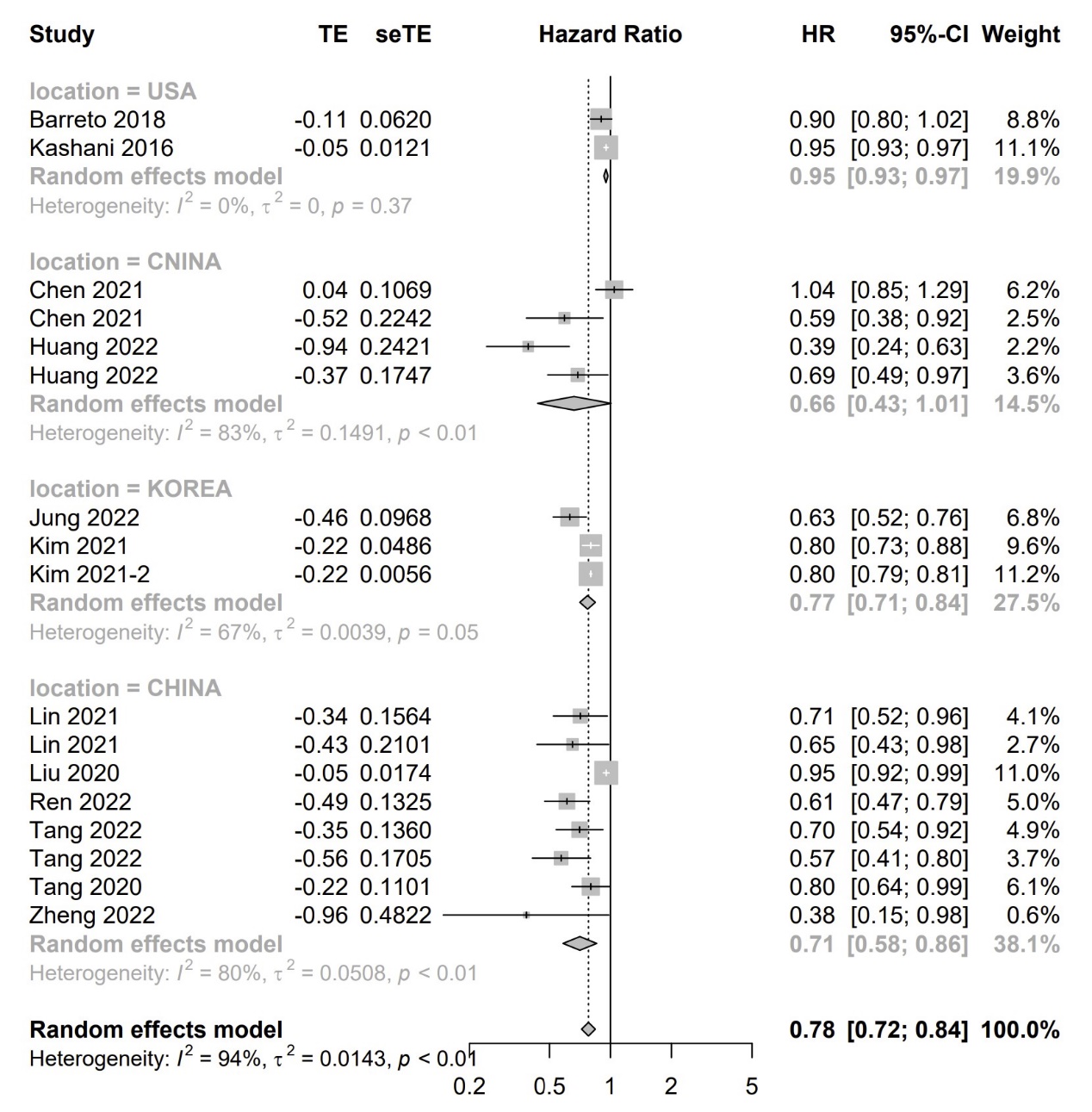


**Fig S3. The forest plot in assessing the impact of CCR on mortality by HR using regression analysis as continuous variables by subgroup of geographic location.**


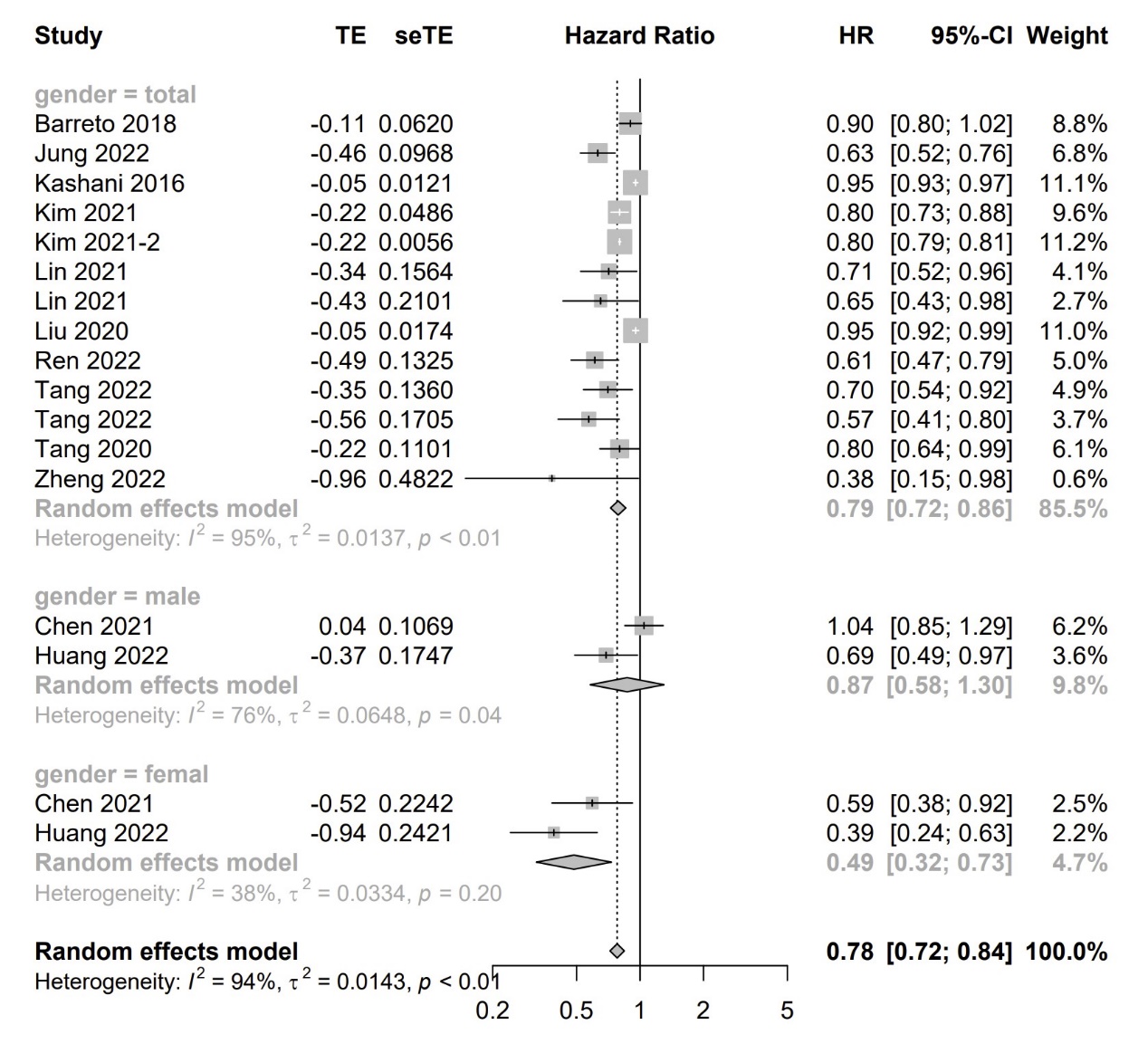


**Fig S4. The forest plot in assessing the impact of CCR on mortality by HR using regression analysis as continuous variables by subgroup of gender.**


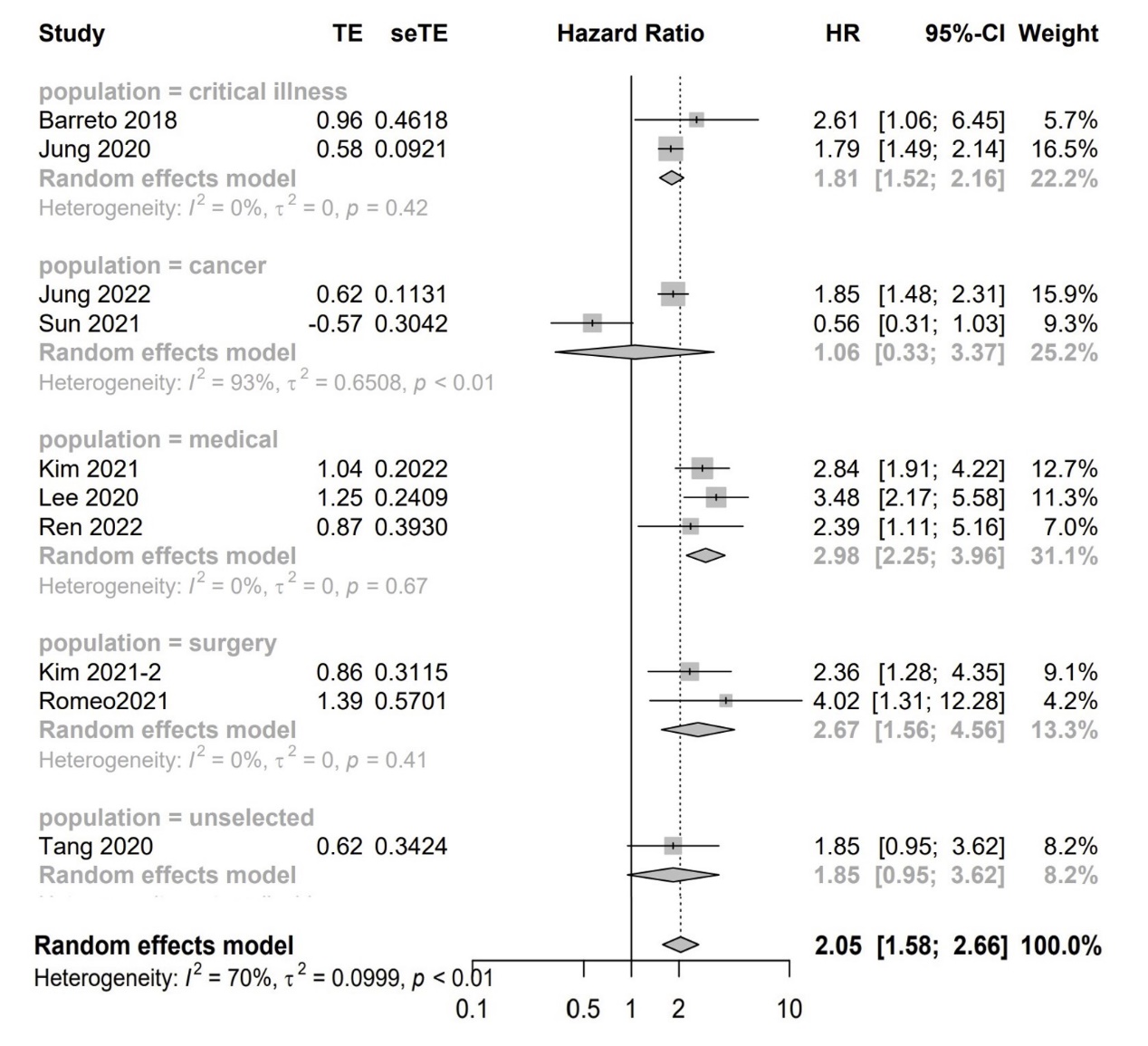


**Fig S5. The forest plot in assessing the impact of CCR on mortality by HR using regression analysis as categorical variables by subgroup of population.**


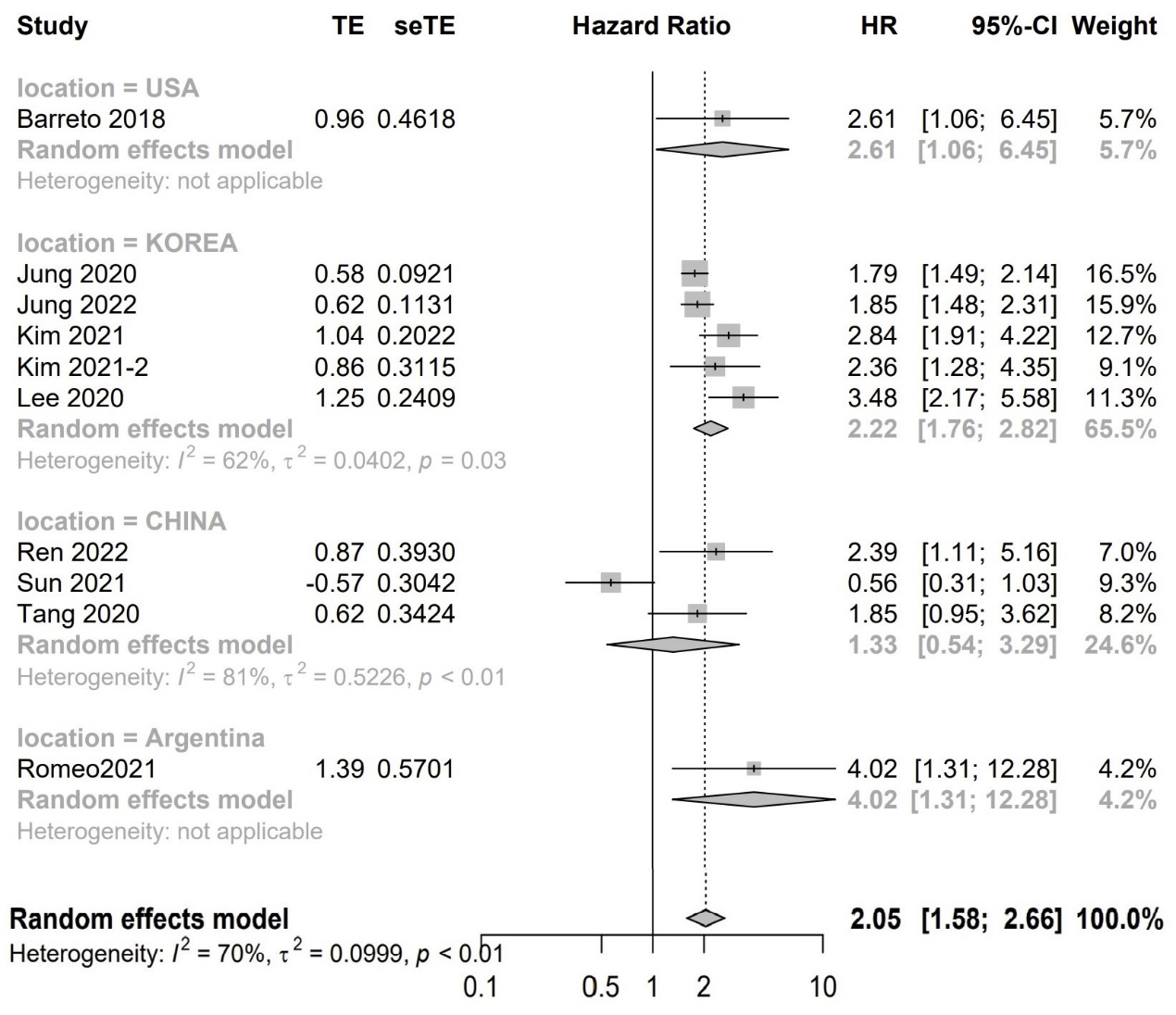


**Fig S6.** **The forest plot in assessing the impact of CCR on mortality by HR using regression analysis as categorical variables by subgroup of geography location.**


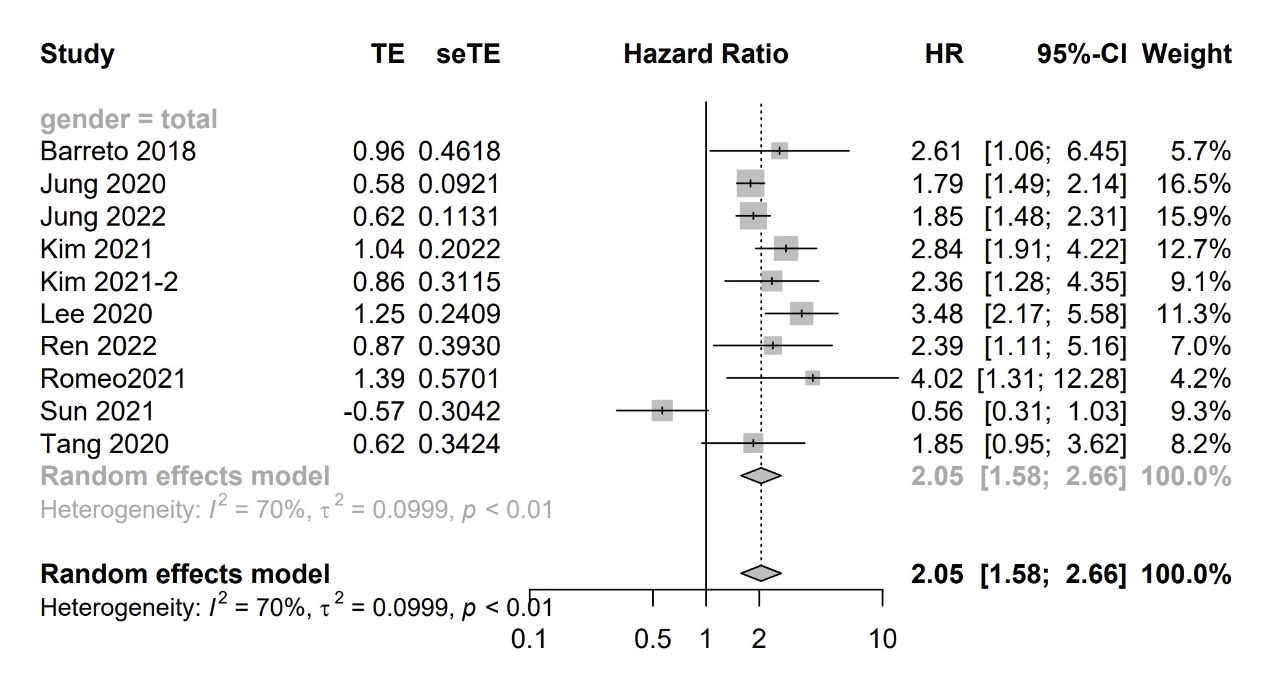


**Fig S7. The forest plot in assessing the impact of CCR on mortality by HR using regression analysis as categorical variables by subgroup of gender.**

**Additional file 5**

**Figure S4:**


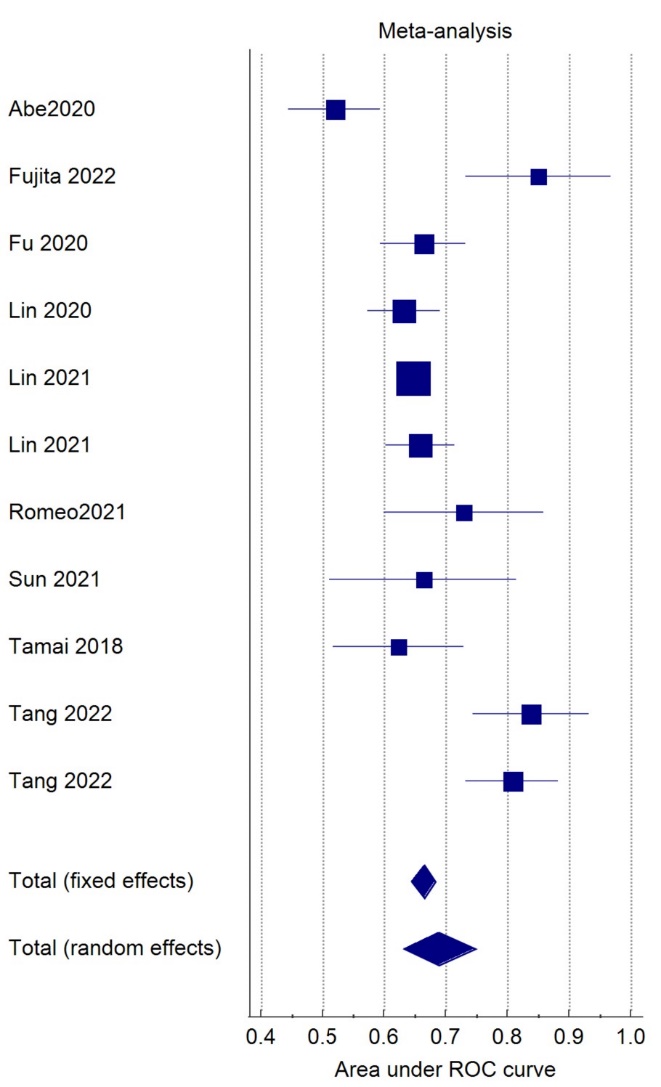


**Fig S8. The pooled estimate of the diagnostic value of CCR for detecting sarcopenia.**

**Additional file 6**


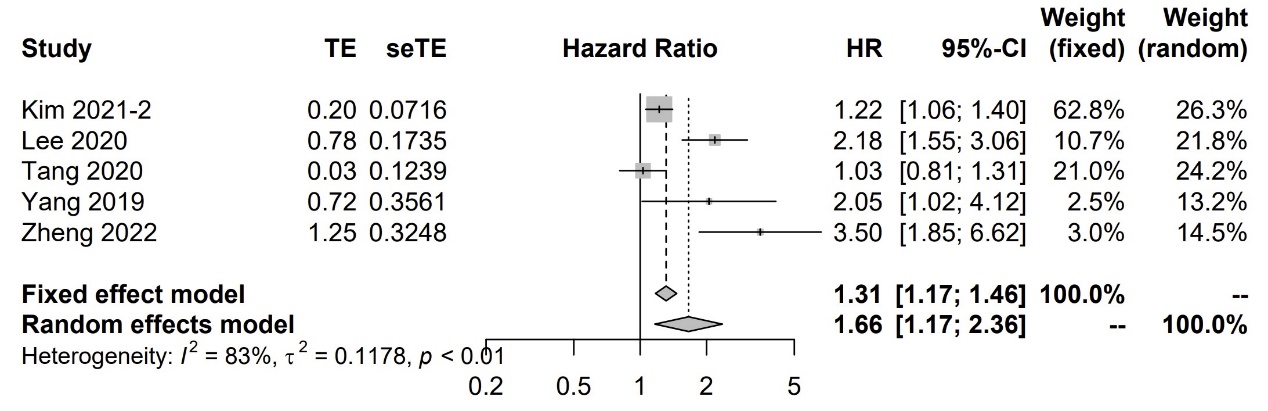


**Fig S9. The pooled estimate of the relationship between serum creatinine/cystatin C ratio and complications.**
